# Heterogeneous fusion of biometric and deep physiological features for accurate porcine cough recognition

**DOI:** 10.1371/journal.pone.0297655

**Published:** 2024-02-01

**Authors:** Buyu Wang, Jingwei Qi, Xiaoping An, Yuan Wang

**Affiliations:** 1 College of Computer and Information Engineering, Inner Mongolia Agricultural University, Hohhot, Inner Mongolia, China; 2 Key Laboratory of Smart Animal Husbandry at Universities of Inner Mongolia Autonomous Region, Inner Mongolia Agricultural University, Inner Mongolia, China; 3 College of Animal Science, Inner Mongolia Agricultural University, Hohhot, Inner Mongolia, China; Vellore Institute of Technology: VIT University, INDIA

## Abstract

Accurate identification of porcine cough plays a vital role in comprehensive respiratory health monitoring and diagnosis of pigs. It serves as a fundamental prerequisite for stress-free animal health management, reducing pig mortality rates, and improving the economic efficiency of the farming industry. Creating a representative multi-source signal signature for porcine cough is a crucial step toward automating its identification. To this end, a feature fusion method that combines the biological features extracted from the acoustic source segment with the deep physiological features derived from thermal source images is proposed in the paper. First, acoustic features from various domains are extracted from the sound source signals. To determine the most effective combination of sound source features, an SVM-based recursive feature elimination cross-validation algorithm (SVM-RFECV) is employed. Second, a shallow convolutional neural network (named ThermographicNet) is constructed to extract deep physiological features from the thermal source images. Finally, the two heterogeneous features are integrated at an early stage and input into a support vector machine (SVM) for porcine cough recognition. Through rigorous experimentation, the performance of the proposed fusion approach is evaluated, achieving an impressive accuracy of 98.79% in recognizing porcine cough. These results further underscore the effectiveness of combining acoustic source features with heterogeneous deep thermal source features, thereby establishing a robust feature representation for porcine cough recognition.

## Introduction

Non-contact, stress-free health behavior detection in pigs is essential for smart farming, and pig interaction behavior recognition is essential for early disease diagnosis [1]. Cough serves as an early sign of respiratory disease in pig houses, making its monitoring essential for the early detection and treatment of such conditions [2,3]. However, relying on manual detection by resident veterinarians leads to delayed response times, substantial costs, and subjective results [4,5]. To address these challenges, many researchers have explored non-contact automatic recognition techniques for identifying pig cough, with a specific focus on pig cough sound recognition [6,7]. In addition, efforts have been made to improve the accuracy and robustness of porcine cough recognition by using infrared cameras and other sensors to capture physiological responses during coughing or observable external features associated with pig physiology [8,9]. These studies mainly focus on porcine cough physiology recognition.

Cough sound recognition has emerged as a low-cost, convenient, efficient, and non-invasive method for monitoring pig health. To enhance classification performance and robustness, researchers have explored various techniques tailored to different environments and application scenarios. These techniques have shown promising results in the field of cough sound recognition. Specifically, one approach involves utilizing machine learning algorithms to extract representative sound features from the time domain, frequency domain, and Mel frequency cepstrum domain. These features are then fed into a classifier for classification [10,11]. Another approach leverages convolutional neural networks to extract deep features from sound spectrograms, enabling effective classification [12,13]. A third approach combines the strengths of the previous two methods through multi-feature fusion classification [14,15]. These three approaches have demonstrated excellent performance in the task of cough sound recognition. However, enhancing representative acoustic features remains a bottleneck challenge, especially in field situations where homogeneous acoustic features make it difficult to achieve optimal classification results, whether using early fusion-feature fusion or late fusion-classifier fusion [16,17]. To overcome this challenge, researchers aim to break the performance bottleneck of cough sound classification by fusing heterogeneous features within the pig pen.

Cough physiological identification techniques leverage infrared cameras or other sensors to capture the physiological responses of pigs when they cough, along with observable external physiological features, to accurately identify coughs [18]. Among the existing methods, the development of physiological image sensors and the use of infrared cameras to capture physiological image data have been explored. Additionally, using Convolutional Neural Networks (CNN) to extract deep physiological features with higher accuracy from captured data with fewer parameters [19,20] has become the state of the arts for cough identification [21]. The development of physiological sensors, however, can be invasive and expensive [22]. As a result, the utilization of CNN to extract deep physiological features has gained traction due to its effectiveness. CNNs have been successfully applied to cough physiological recognition in two primary ways. One is an end-to-end approach for homogeneous physiological image classification tasks [23,24]. However, this approach faces the same performance bottleneck as homogeneous sound classification. The other approach extracts deep physiological features from the CNN and performs multimodal feature fusion with other heterogeneous features (e.g., biometric features). The fused feature vectors are then input into a lightweight classifier to complete the classification task [25,26]. In other words, the various improved CNN functions as a deep physiological feature extractor that can reduce computational costs to accelerate classification efficiency [27]. Moreover, the incorporation of additional dimensions of heterogeneous features helps address the bottleneck of classification performance improvement. Thus, adopting a custom shallow CNN architecture to extract deep physiological features and fusing them with acoustic features to feed a lightweight SVM classifier holds significant promise for porcine cough recognition.

This work aims to combine cough acoustic features with cough physiological features, taking into account the pathology of cough. The objective is to construct a robust and effective feature representation for porcine cough in pig housing by combining heterogeneous multidimensional fusion feature signals consisting of cough sound biosignal and cough body temperature infrared signal. This integration is expected to improve the performance of porcine cough recognition significantly. To achieve this, a three-stage approach is proposed. Firstly, acoustic features are extracted from the cough sound, and the SVM-RFECV algorithm is employed to select the optimal acoustic source biometric features. Secondly, a custom CNN is used to extract thermal source deep physiological features from infrared thermal images, and the feature vectors of the two fully connected layers are fused to obtain layer fusion deep physiological features. Lastly, the acoustic source biometric features are early fused with the thermal source deep physiological features and layer fusion deep physiological features, respectively. These fused features are then fed into a lightweight SVM classifier to complete the classification task.

In summary, this study makes the following contributions:

1. Proposal of a novel framework: This study introduces a novel framework that combines acoustic source features with deep physiological features for pig cough recognition.
2. Development of a feature selection method: To optimize the recognition process, a feature selection method is proposed to extract a representative set of acoustic features of pig coughs.
3. Construction of a CNN architecture: A carefully designed CNN architecture is constructed to extract deep physiological features from thermal images to enhance the recognition performance of pig coughs.
4. Comparative analysis with existing models: The proposed method outperforms existing CNN models in terms of recognition speed, model size, and classification performance.

The remainder of this paper is organized as follows. Materials Section provides the work related to the materials. In Methods Section, the methods involved in the experiments are comprehensively described. The results obtained from our experiments are presented in Experiments and Results Section. Discussion Section offers a discussion of the results. Finally, conclusions are drawn in Conclusions Section.

## Materials

### Housing and laboratory animals

**Fig 1:**
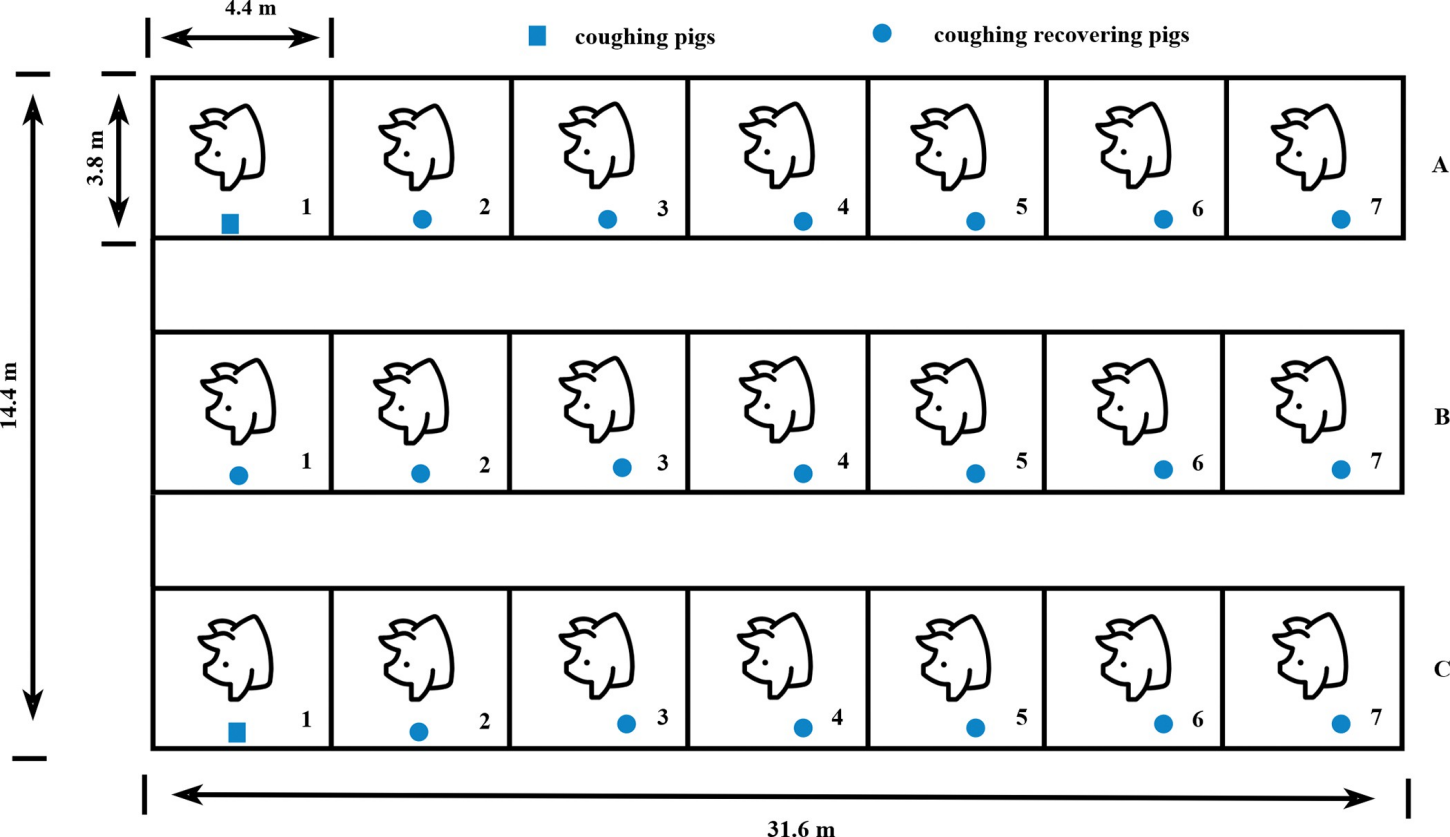
Layout of the experimental pig house.

The data used in this study were collected in a real farming environment in the isolation barn of a large commercial fattening pig farm in Hohhot, Inner Mongolia Autonomous Region, China. The fattening farm has a production scale of 100,000 hogs and raises three breeds, including Large White, Long White, and Duroc, and abnormally sick pigs are sent to the isolation barns for isolation. One of the isolation barns with a capacity of 218 pigs was selected for this experiment. The resident veterinarians isolated pigs of different breeds, age groups, and coughing conditions into the isolation barn was selected. The experiment lasted for 15 days, and the pigs in the isolation barn were constantly transferred in/out of the barn in a dynamic and real-time manner to ensure complete coverage of pigs with different conditions. The experimental isolation barn comprises three units, A, B, and C, each containing seven pens. A1 and C1 house the coughing pigs for the experiments (up to 14 pigs per pen), and A2, B1, B2, and C2 hose the recovering pigs for the experiments (occasional coughing or no coughing, up to 10 pigs per pen), with pens next to each other and interacting with each other in order to reproduce the real environment (coughing) in the production barn. The pens are located next to each other and interact, completely reproducing the real environment in a production barn (coughing pigs surrounded by non-coughing pigs, interacting with each other). The layout of the experimental pigs and pens is shown in Fig 1.

### Data acquisition and preprocessing

**Fig 2:**
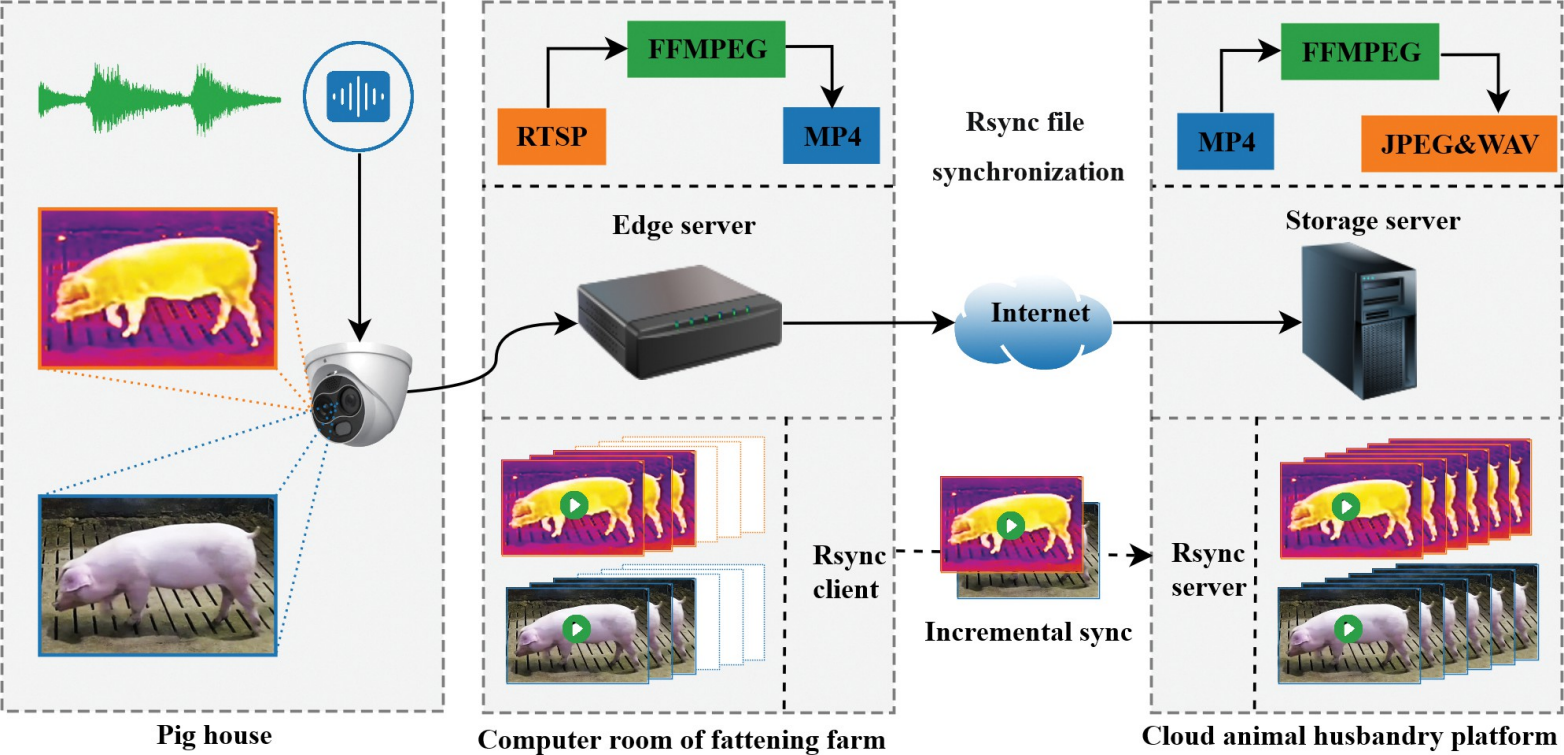
Multimodal data acquisition system based on cloud-edge-end architecture.

The data collection system uses a real production system deployed in the experimental pig house and adopts a cloud-edge-end architecture. The cloud terminal of this experiment includes a pickup for collecting cough sounds and a camera for infrared data deployed in the pig house. The camera and pickup are consistently gathering audio and video data within the pig house, operating 24/7. The camera is a binocular dome camera (TB-1217A-3/PA, Hikvision, Hangzhou, China. Visible light: 25FPS, 4096Kbps, H.264. IR: 1280×720, 25fps, 4096Kbps, H.264 coding), the camera is fixed on a 2-meter-high wall on one side of the pigsty, with an adjustable viewing angle, which makes it easy to cover the whole pigsty. The pickup was an audio recorder (DS-2FP4121-OW-AI, Hikvision, Hangzhou, China, 48kHz, 64kbps, AAC). The cameras and pickups are connected with special audio/video adapter cables. In this way, the installation position of the equipment can be flexibly selected according to different pigsty environments, and the distance between the camera and the pickup can be flexibly adjusted. The edge server is a desktop PC server (OptiPlex 3070, Dell, USA) located within the pig farm’s server room, establishing a seamless connection with the cameras via Ethernet. Through the utilization of the FFMPEG software, the Edge server effectively captures real-time feeds from both the visible light channel (1920×1080) and thermal imaging channel (1280×720) using the RTSP protocol. Real-time acquisition to the server hard drive, respectively, saved as the acquisition start time as the file name of the mp4 file, i.e., visible light is named as “acquisition start time _ camera number.mp4” and thermal imaging is named as “acquisition start time _ camera number _ tf.mp4”. Importantly, each mp4 file contains audio data and generates new audio and video files every 10 minutes. To facilitate synchronized data transfer between the Edge server of the pig farm and the Storage server of the cloud animal husbandry platform [28], a robust Internet connection links the two. The Rsync file synchronization protocol plays a pivotal role in automatically and incrementally transferring audio and video files from the pig farm to the cloud animal husbandry platform. Upon successful synchronization, files are automatically deleted from the Edge server. The Storage server within the cloud animal husbandry platform employs FFMPEG software once again to extract audio from the received visible light audio and video files. These audio segments are then stored as a.wav file, adopting the corresponding video file’s name format (“Acquisition Start Time_Camera No.wav” and “Original Audio and Video File Name.wav”). The data acquisition system used in this study is a real production data acquisition system with good end-to-end generalization capability. Finally, 2160 original data sets were collected over 15 days using a cloud-edge-end data acquisition system. Each set comprises a 10-minute audio WAV file and its corresponding 10-minute infrared video MP4 file. The architecture of the data acquisition system is shown in Fig 2.

For the 2160 sets of raw data files collected, the lens occlusion blurred frames, feeding, immunization, treatment, and other feeding activity-related frames in the raw data were eliminated, and finally, 135 hours of valid audio (WAV) and video files (MP4) were obtained. The visible video is in RGB mode during the day and converted to grayscale mode at night, which is used to help experts in data annotation. The image palette mode for the thermal imaging video was set to "Iron Red".

**Fig 3:**
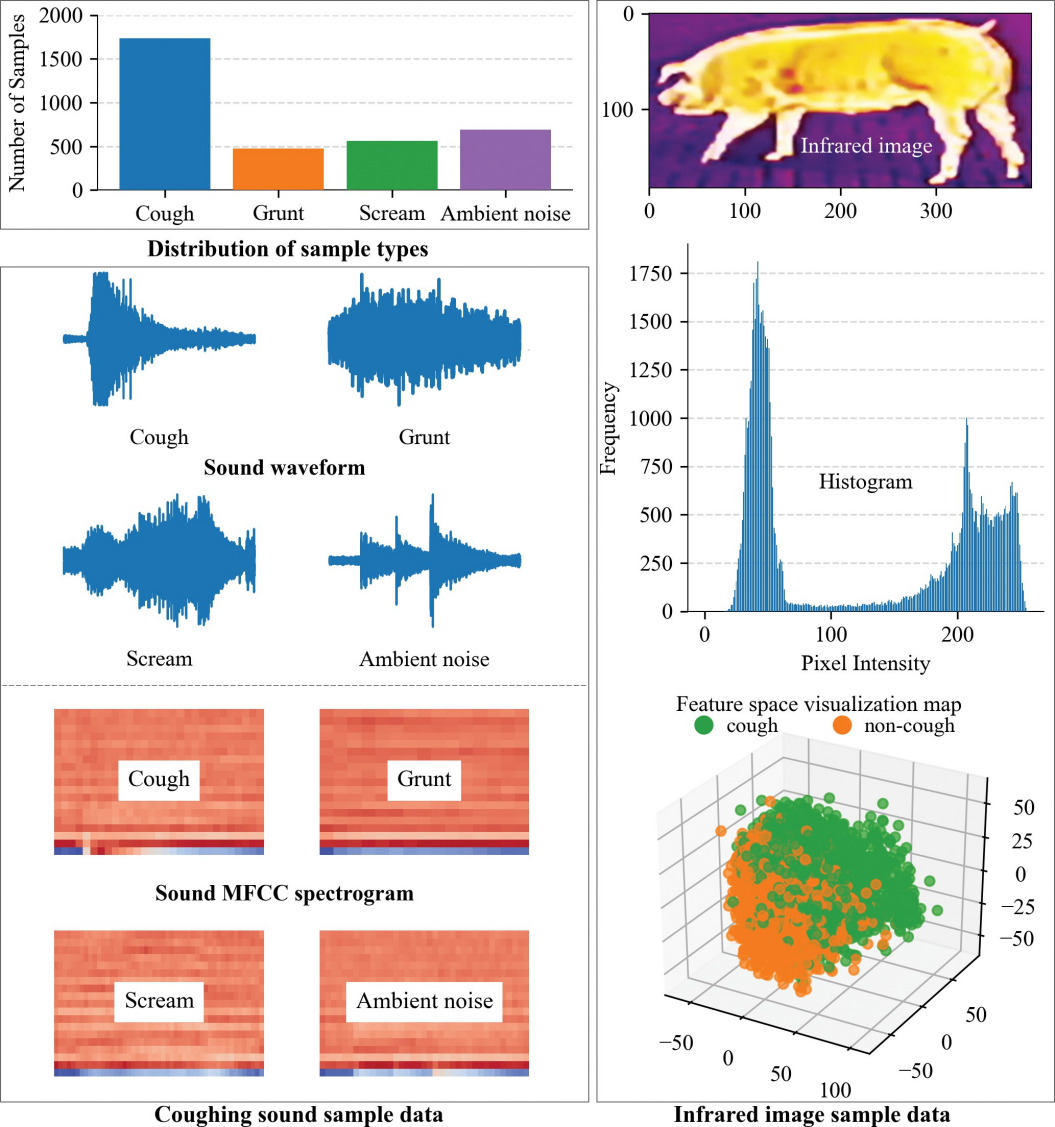
The data distribution graph.

Leveraging the versatile Librosa tool [29], specific audio segments were meticulously extracted from the WAV files, applying predefined conditions (volume threshold ≥ 33dB, length threshold ≥ 2s). The nomenclature of these WAV files was crafted to incorporate the commencement timestamp, enhancing organizational clarity. Subsequently, the WAV file dataset underwent a clustering process utilizing the K-means algorithm. This algorithm, applied to the audio cough data, facilitated the initial identification of clusters associated with coughing events. To refine and classify these clusters, a 10th-order Butterworth filter with a cutoff frequency ranging from 100Hz to 16kHz was employed. The resultant one-dimensional filtered sound data was then transformed into two-dimensional Mel-Frequency Cepstral Coefficients (MFCC) data, enhancing the representation of acoustic features. Further refinement ensued through the application of the K-means algorithm, selectively isolating clusters relevant to coughing sounds. A collaborative effort involving subject matter experts, resident veterinarians, and aided by visible video cues, led to the manual labeling of each audio file based on the clustering results. The manual annotation encompassed diverse labels such as coughs, squeals, grunts, feeding sounds, human voices, door movements, chain sounds, and other ambient noise types. In the final steps of data preprocessing, video clips were systematically extracted from corresponding infrared video files, guided by the timestamps of the manually labeled sound clips. The culmination of these meticulous processes yielded a cohesive dataset of paired cough sound (WAV) and cough infrared (MP4) files. The standardized naming format for these files incorporated essential details, including the original audio/video file name, audio start timestamp, audio duration time, and a sequence number. The data distribution is shown in Fig 3.

## Methods

### Methodological overview

**Fig 4:**
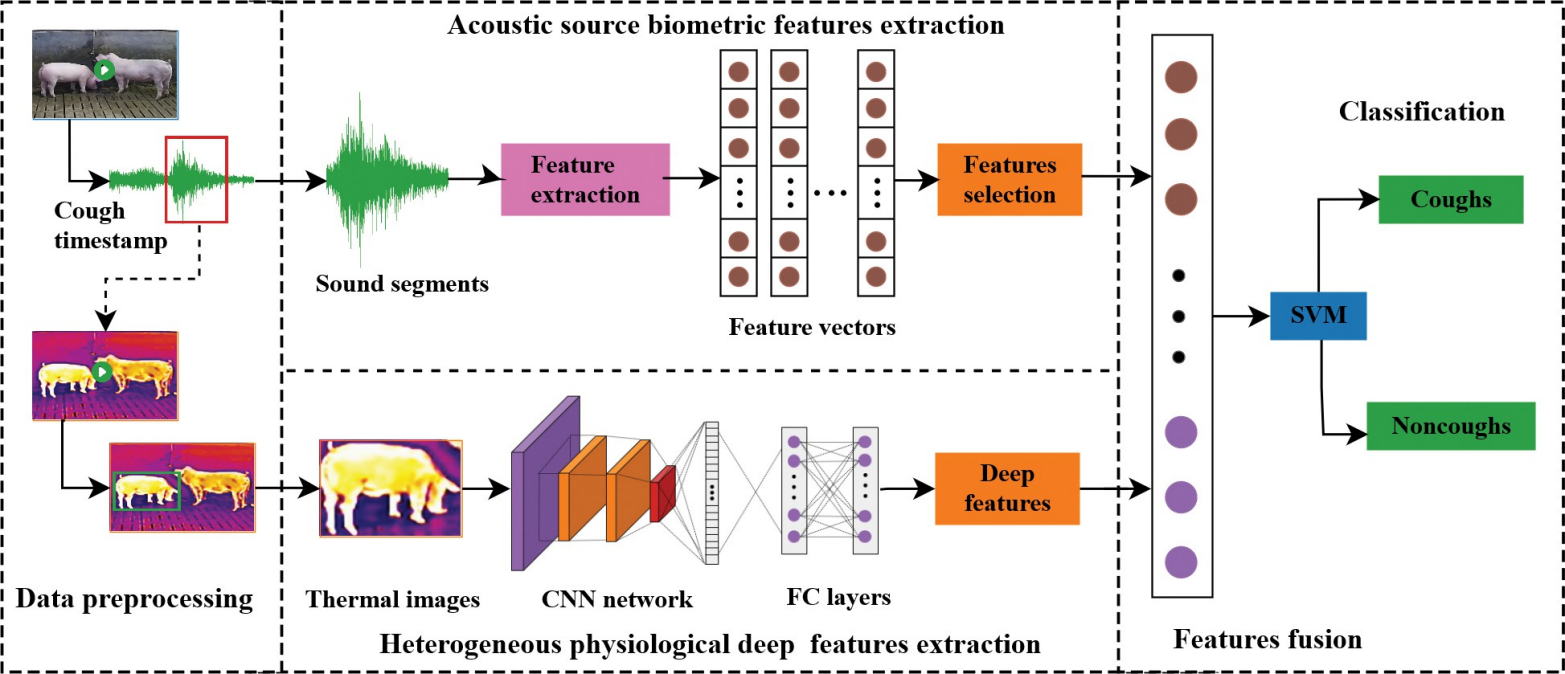
The flowchart of the proposed porcine cough recognition.

Coughing behavior, by its apparent features, produces an acoustic signal. From a physiological point of view, it will be accompanied by a change in body temperature, which will produce physiological signals. Therefore, this paper considers the influence of these two factors in recognizing cough. At the same time, the acoustic signal reflecting the biological characteristics and the deep features reflecting the physiological characteristics of body temperature are collected, and the alignment is integrated early to improve cough recognition performance. This study introduces a novel approach that utilizes significantly differentiated heterogeneous features to effectively perform automatic porcine cough identification. The flowchart of the proposed method is illustrated in Fig 4. Firstly, acoustic source biometric features are extracted from the preprocessed audio clips essential characteristics of the cough sounds. Secondly, thermal source deep physiological features, which reflect the distribution of body temperature, are extracted from thermal images using a lightweight shallow CNN. Thirdly, the extracted acoustic and physiological depth features are aligned and concatenated through early fusion to provide distinguishable multi-source heterogeneous fusion features. Finally, an SVM classifier is exploited to complete the classification of coughs and non-coughs.

### Acoustic source biometric features

Using the audio processing method of the Librosa library, each sample of the cough audio clip in our constructed dataset was used as input, from which 29 acoustic features were extracted, and then recursive feature elimination combined with a cross-validation algorithm was used for feature selection, to select the acoustic source biometric feature to represent the cough sound signal. Time-domain analysis captures features in terms of time, frequency-domain analysis reveals features in terms of frequency, and cepstrum-domain analysis provides information on acoustic properties. Combining these results enables the extraction and classification of cough sounds, facilitating automated detection and recognition. In the time-domain analysis, the time-domain statistical features such as mean, variance, and energy are calculated to understand the temporal characteristics of cough sounds. Frequency domain analysis provides the energy distribution of the cough sound over different frequency components, thus revealing the frequency characteristics like frequency components and frequency range. Cepstrum domain analysis is a special frequency domain analysis method for extracting the acoustic characteristics from signals. Cepstrum domain analysis can provide the resonance characteristics of cough sounds, which helps to distinguish different types of cough sounds. By combining the analyses of the time domain, frequency domain, and cepstrum domain features, the characteristics of cough sounds can be revealed in a comprehensive way. The combinations of 29 acoustic source features consisting of the time, frequency, and cepstrum domains were extracted from the one-dimensional sound signal. The time domain features include Root Mean Square (RMS) energy and Zero Crossing Rate (ZCR) [30]. The frequency domain features include spectral envelope, constant Q cepstral coefficients (CQCC), spectral centroid, spectral bandwidth, spectral rolloff, spectral flatness, and spectral flux [31]. Also, we extracted twenty MFCC cepstrum domains [32].

Feature selection assumes paramount importance, especially within the realm of high-dimensional datasets like those characterizing coughing speech data. Prudent feature curation bolsters model interpretability, curbs overfitting tendencies, enhances computational efficiency, and ultimately elevates the precision of coughing event classification.

Support Vector Machine Recursive Feature Elimination Cross-Validation (SVM-RFECV) stands as a cutting-edge feature selection technique specifically tailored for the classification of coughing events utilizing speech data. This novel methodology synergizes the capabilities of Support Vector Machines (SVM) and Recursive Feature Elimination Cross-Validation (RFECV), as outlined by [33]. By iteratively eliminating noise data unrelated and redundant features through feature selection, we aim to improve the SVM model’s prediction accuracy and generalization ability [34].

Within the domain of coughing speech data, the potency of SVM-RFECV derives from the incorporation of SVM as the foundational classifier. SVMs are renowned for adeptly handling high-dimensional data and adeptly capturing intricate patterns. By entwining SVM with RFECV—a recursive feature elimination mechanism—SVM-RFECV systematically sifts and preserves the most pertinent attributes for classifying coughing events, eliminating irrelevant noise data (such as sustained high-frequency audio signals).

The ingenuity of SVM-RFECV hinges on its iterative feature pruning procedure, meticulously devised for the nuances of coughing speech data. The RFECV algorithm assesses feature importance through weight assignment during each iteration. Weight calculations, as delineated by Formula (1), coupled with the computation of importance levels as per Formula (2), facilitate the ranking of features. Subsequently, features with lower weights, indicative of diminished relevance, are progressively excised from the feature pool. The SVM model is then retrained using the winnowed feature ensemble. This iterative sequence perseveres until an optimum assemblage of features, germane to the classification of coughing events, is achieved [35].

$$w_{i}=|\alpha_{i}\times y_{i}|,i\in [1,P],$$ (1)

$$I(f_{i})=n-k_{i}$$ (2)

where *f*_i_ denotes the ith feature in the dataset, w_i_ denotes the weight of the ith feature, P denotes the number of features (where P is set to 29), y_i_ denotes the classification class of the ith feature, *I*(*f*_i_) denotes the importance of the ith feature, n denotes the number of samples, and *k*_i_ denotes the number of times the ith features are removed in the recursive feature elimination process. This process removes redundant acoustic features through sound source feature selection. The highest predicted F1 score achieves the optimal feature combination to generate a new set of acoustic source biometric features.

### Thermal source deep physiological features

The occurrence of coughing behavior in porcine respiratory infections triggers discernible alterations in the infrared body temperature signal of the pigs. Particularly noteworthy is the distinctive variance in body temperature between coughing pigs and their healthy counterparts. Coughing pigs manifest elevated body temperatures that exhibit unique infrared signal patterns. This observation underpins the efficacy of a method involving the acquisition of infrared images from coughing pigs, extraction of infrared deep features using a Convolutional Neural Network (CNN), and feeding these features into an SVM classification network. The outcome is a non-contact, automated system capable of detecting pig coughing events.

Physiological deep features, or high-level features beyond epistemic knowledge, are derived through deep learning techniques applied to raw thermal images of physiological signals. These features hold immense potential across various applications, including diagnostics and disease prediction [36]. In our study, we harnessed a lightweight shallow CNN architecture to extract infrared deep physiological features that faithfully mirror real-time surface temperatures of monitored pigs.

The integration of infrared thermal images within our approach is motivated by two pivotal factors. Firstly, infrared thermal images aptly capture the distribution of body surface temperatures while encapsulating the physiological nuances of living organisms [37]. Their efficacy in automated porcine cough event identification has been well-documented [38]. Secondly, the shallow CNN architecture proves well-matched for feature extraction from thermal images [39]. The amalgamation of this architecture with SVM classification engenders highly accurate classification outcomes while significantly boosting execution speed [40].

Our methodology encompasses the gathering of infrared images from coughing pigs, the extraction of infrared depth features via a CNN network, and their subsequent input into an SVM classification network. This non-contact approach translates into an automated mechanism for detecting pig coughing, thereby delivering an exhaustive and robust analysis of the physiological attributes associated with coughing events.

Different shallow convolutional neural networks have their own strengths. LeNet-5 is a classical convolutional neural network model proposed by Yann LeCun et al. in 1998, which may not effectively capture higher-level, more abstract feature information for complex image classification tasks [41]. AlexNet was proposed by Alex Krizhevsky et al. in 2012 and uses the ReLU activation function to nicely mitigate the gradient disappearance problem and performs well in large-scale image classification tasks but is prone to overfitting and requires significant time and resources for training and inference [42]. Compared to other deeper architectures such as VGG [43] and ResNet [44], where LeNet-5 is a shallow network more suitable for smaller datasets, AlexNet is a deeper network for extracting higher-level feature information. Our experiments are on a private and limited dataset where higher-level deep features must be extracted for evaluation. The deployment requires an optimal solution between recognition speed, model size, and performance.

**Fig 5:**
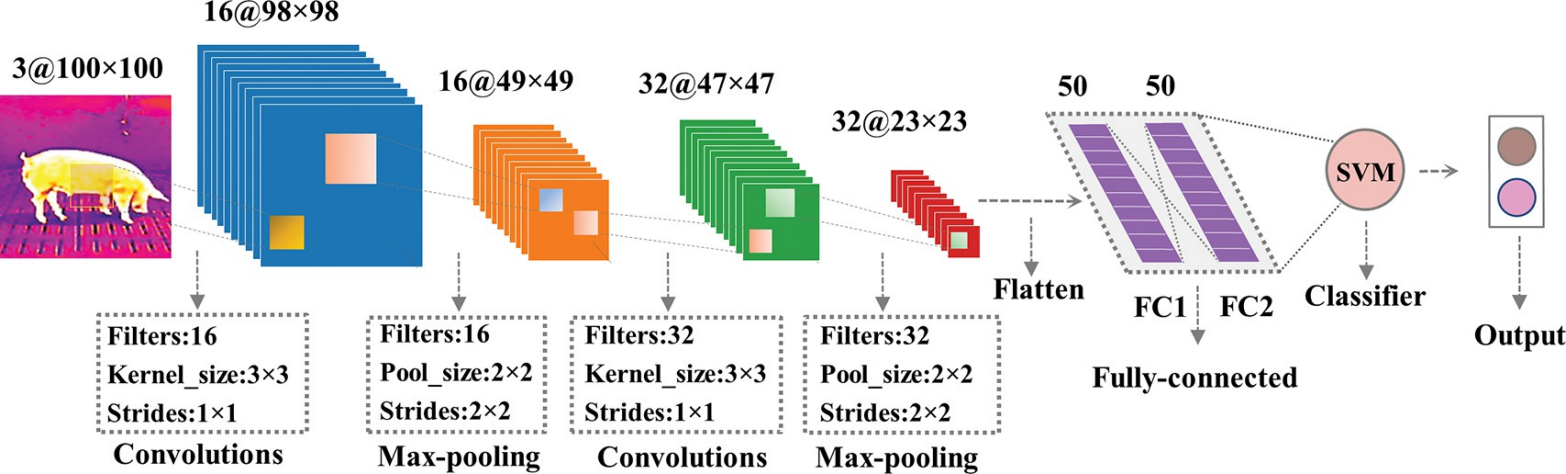
ThermographicNet for extracting thermal source deep physiological features.

Therefore, we propose a lightweight shallow convolutional neural network architecture, named ThermographicNet, as a deep physiological feature extractor. ThermographicNet combines the advantageous design elements of the Lenet-5 and Alexnet architecture, as illustrated in Fig 5.

The proposed ThermographicNet architecture incorporates specific design choices to effectively process thermal images for porcine cough recognition. The network comprises several key layers and operations, as described below:

1. Input layer: The network takes a 100×100 pixel thermal image as input.
2. Convolutional layers: The first convolutional layer consists of 16 filters with a size of 3×3, and the second convolutional layer includes 32 filters of the same size. Both convolutional layers apply Rectified Linear Unit (ReLU) activation functions, which enables faster convergence and avoids gradient vanishing.
3. Max-pooling layers: Two max-pooling layers with a size of 2×2 follow the convolutional layers. These layers downsample the spatial dimensions of the feature maps, capturing the most salient information while reducing computational complexity.
4. Flattening layer: The output of the previous layer is flattened into a one-dimensional vector.
5. Dropout layer: It is designed to randomly deactivate a fraction of neurons with a probability of 0.5 to alleviate the overfitting problem by enhancing the network’s generalization capability.
6. Fully Connected (FC) layers: Two FC layers with 50 neurons each are connected to the Dropout layer.

2D convolution was chosen to convolve the different thermal physiological features simultaneously as the thermal distribution of the thermal image is a 3-channel 2D image. ReLU was chosen because it converges faster and does not suffer from gradient disappearance. The Dropout layer was chosen to increase the generalization capability of the network in order to prevent possible overfitting problems for thermal images extracted within a small time slice (≈2.13s) of a persistent cough. Adam was chosen as the optimizer for model training, Sparse_Categorical_Crossentropy was chosen as the loss function, and accuracy was used as the model evaluation metric.

The performance of two fully connected layers of Lenet-5, AlexNet, DenseNet121, Vgg16, Vgg19, ResNet50, ResNet101, ResNet152, and our proposed ThermographicNet network are first evaluated separately for deep physiological feature extraction. Then, the two fully connected layer feature vectors extracted from the ThermographicNet network were aligned and concatenated to form the layer fusion deep physiological features for classification evaluation. Finally, the FC1 feature vector extracted from the ThermographicNet network was selected as the thermal source of deep physiological features.

### Feature fusion methods

**Fig 6:**
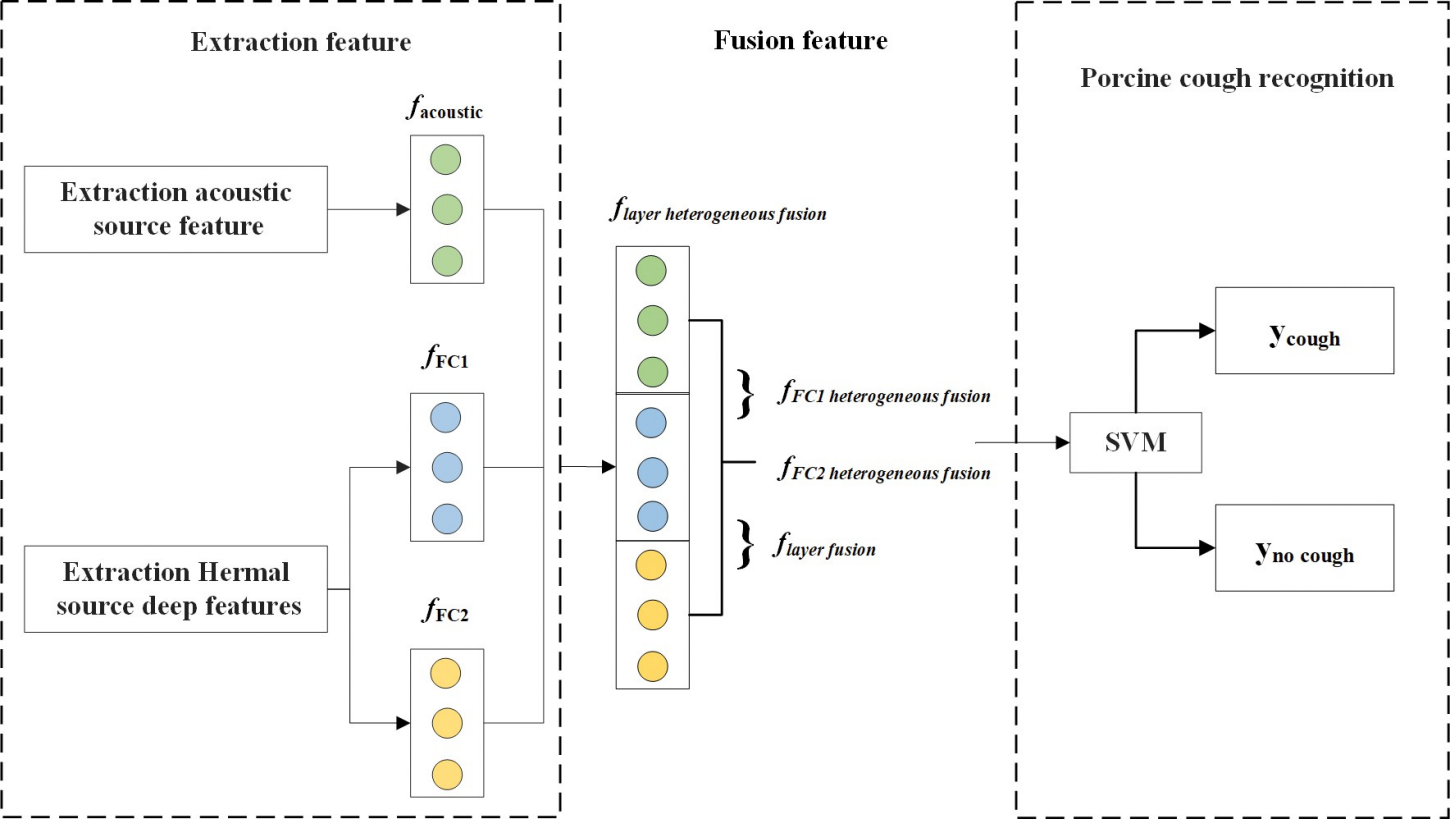
Flowchart of early feature fusion technique with four different fusion strategies.

Extracting acoustic biometric features and deep physiological features from audio and infrared data, respectively, and fusing them to provide distinguishable multimodal fusion features for accurate automatic cough recognition to break through the performance bottleneck of cough sound classification. The fusion method adopts feature-level fusion, also known as early fusion, which is the most commonly used strategy in multimodal recognition systems. After extraction, it means immediately connecting features extracted from different modalities into a single high-dimensional feature vector. We have provided four different fusion strategies to evaluate the cough classification task. Specifically, first, obtain the acoustic source biometric features (*f*_*acoustic*_) from the acoustic source, the deep physiological features (*f*_*FC1*_) from the first fully connected layer of the ThermographicNet network, and the physiological deep feature (*f*_*FC2*_) from the second fully connected layer. Then, concatenate *f*_*FC1*_ and *f*_*FC2*_ to obtain *f*_*layer fusion*_, concatenate *f*_*acoustic*_ and *f*_*FC1*_ to obtain *f*_*FC1 heterogeneous fusion*_, concatenate *f*_*acoustic*_ and *f*_*FC2*_ to obtain *f*_*FC2 heterogeneous fusion*_, and concatenate *f*_*acoustic*_ and *f*_*layer fusion*_ to obtain *f*_*layer heterogeneous fusion*_. Finally, input these features into an SVM classifier to obtain the classification results for the cough task, as shown in Fig 6.

### Classification

In the porcine cough recognition classification task, selecting an appropriate classifier is crucial to handling the extracted features effectively. The early fusion technique is employed to extract highly representative nonlinear and high-dimensional feature vectors that capture the essential features of cough from multiple modalities. These feature vectors are then concatenated and used as input for the classifier. Considering the requirements of handling high-dimensional feature spaces and nonlinear decision boundaries, the SVM (Support Vector Machine) classifier emerges as an advantageous choice.

The SVM classifier possesses the necessary strengths to handle the complexities of the cough recognition task. It excels in effectively processing high-dimensional feature spaces and modeling nonlinear decision boundaries. Leveraging these capabilities, the SVM classifier accurately classifies cough events even in intricate and overlapping patterns. By utilizing the SVM classifier, we enhance the performance and reliability of the cough recognition system, contributing to the advancement of automatic cough identification technology.

## Experiments and results

The experimental datasets are constructed in this Section, and each experiment’s model evaluation metrics, implementation method, main execution environments configuration, and results are given.

### Datasets

We will create our dataset using the preprocessed pig cough audio files and thermal imaging video files obtained from Data acquisition and preprocessing Section. We load the corresponding thermal imaging video files based on the cough audio file names. We extract the thermal image video clip from each thermal imaging video file. A Python script is used to extract five frames of image data from each video clip, which serve as candidate thermal source images.

With the assistance of the visible video and the resident veterinarian, the expert selects one image from the candidate thermal source images as the fusion target image. The selected fusion target image is then labeled using the LabelMe software [45]. The extracted thermal image file is named based on the corresponding audio file and marked as a positive sample.

Similarly, the expert follows the same procedure for negative samples to select the target pig image as the thermal image corresponding to the non-cough audio. We extract 1736 cough sounds and their corresponding 1736 physiological thermal images, which form the positive sample set. The negative sample set also consists of 1731 non-cough audio fragments (477 grunts, 565 screams, and 689 ambient noises) and their corresponding 1731 thermal images.

### Metrics

The dataset is divided into a 70% training set and a 30% test set with no duplicate samples between the two sets. To evaluate the performance of our proposed method, a ten-fold cross-validation method with grid search is utilized on the training set. It explores and determines the optimal set of hyperparameters for training the SVM algorithm. The initial values of the hyperparameters are set to set with default values, and the corresponding models are generated based on the preprocessed training set. The test set is then used to evaluate the performance of the best pre-trained model obtained from the grid search. This grid search procedure allows us to systematically explore different combinations of hyperparameters and select the ones that yield the best performance for our cough recognition task. In the grid search process, for linear models, C is set to 0.9 and 1. For nonlinear models, the radial basis function (RBF) is used as the kernel function, and the value of Gamma is computed by Formula (3) [46], with the value of C set to the same value as the linear models.

$$Gamma=\frac{1}{n\_features\times X.\mathrm{var}()}$$ (3)

where X represents the training sample, *n_features* stands for the number of features in the input sample, and var() means the variance of the training set samples.

After training the model, its classification performance is evaluated using the test set. The evaluation metrics used for our model include accuracy, recall, precision, and F1-score (see Formulas (4)–(7). Here, we define cough as a positive sample and non-cough as a negative sample. True positives (TP) refer to correctly classified cough samples, true negatives (TN) represent correctly classified non-cough samples, false positives (FP) indicate non-cough samples misclassified as cough, and false negatives (FN) represent cough samples misclassified as non-cough. The evaluation metrics are calculated as follows:

$$Accuracy=\frac{TP+TN}{\left(TP+TN+FP+FN\right)}$$ (4)

$$\mathrm{Pre}cision=\frac{TP}{\left(TP+FP\right)}$$ (5)

Recall=TP(TP+FN) (6)

F1−Score=2×Precision×Recall(Precision+Recall) (7)

### Implementation

The primary execution environments and parameters configured for our experiments are listed in Table 1. The program is implemented based on the Python language, the LibROSA library, and the Keras deep learning framework.

**Table 1:** Description of main execution environments and parameter configurations.

Execution Environment	Parameter
CPU	Intel Xeon Gold 6139M With 2.30GHz
Memory	251GB
GPU	NVIDIA Corporation GA102 [GeForce RTX 3090] (rev a1)
GPU Memory	24GB
Cuda	11.2
Anaconda	23.1.0
Python	3.9.7
LibROSA	0.9.2
Scikit-learn	1.1.3
Keras Version	2.5.0
Tensorflow-GPU	2.5.0
Optimizer	Adam optimizer
Batch Size	32
Learning_Rate	0.001
MFCC_Dim	20
Dropout_Prob	0.5
Epoch_Max	50
Storage Device	Samsung SSD 870 With 4TB
Operating System	Linux Ubuntu SMP 18.04.1

The automatic framework for pig cough recognition is implemented using the Python programming language. The processing and feature extraction of cough audio data is implemented using the Librosa library. The processing and physiological deep feature extraction of cough infrared image data are implemented using the Keras deep learning library, including the design, debugging, evaluation, application, and visualization of CNN network models. Fusion and classification are implemented using the scikit-learn machine learning toolkit. Grad-CAM (Gradient Weighted Class Activation Mapping) [47] is used to interpret the ThermographicNet model visually.

The cough audio data processing program mainly includes preprocessing, feature extraction, and feature selection of cough data. Preprocessing and feature extraction primarily utilize libraries such as Librosa, NumPy, CSV, and OS. The cough audio has a sampling rate of 48000Hz, followed by the extraction of 29 sound features using functions such as zero_crossing_rate, RMS, chroma_stft, chroma_cqt, spectral_centroid, spectral_bandwidth, spectral_contrast, spectral_rolloff, spectral_flatness, onset_strength, and MFCC provided by the Librosa feature class. In the feature selection, libraries such as scikit-learn, pandas, and matplotlib are mainly used. The data set is split into training and testing sets using the train_test_split method in the model_selection class of the scikit-learn package, with the testing set accounting for 30%. Recursive feature elimination and 5-fold and 10-fold cross-validation are performed using the RFECV function in the model_selection class. Finally, the cough sounds are classified using the SVC function in the SVM class of the scikit-learn library.

Processing cough infrared image data mainly involves network design, debugging, and evaluation. Using libraries such as Keras, scikit-learn, pandas, NumPy, and OS, the infrared image data is resized to 100×100 pixels. The data set is split into training and testing sets using the train_test_split method in the model_selection class of the scikit-learn package, with the testing set accounting for 30%. Subsequently, network models, including Lenet-5, AlexNet, DenseNet121, Vgg16, Vgg19, ResNet50, ResNet101, ResNet152, and ThermographicNet, are designed using the Keras library and trained and evaluated. The optimizer used is Adam, with a maximum number of epochs (epoch_max) set to 100, a batch size of 32, a learning rate of 0.001, and 20% of the training set used as a validation set for network evaluation. After training all the network models, the load_model function in the models class of the Keras library is used to load the models. The deep features of the infrared images are predicted using the models, and the FC layer physiological deep features data are extracted for subsequent deep feature fusion.

The scikit-learn, Keras, pandas, and NumPy libraries are primarily used to perform three fusion experiments in the feature fusion and classification experiments. The fusion function is implemented using the concat function provided by the pandas library. Classification is performed using the SVC function in the SVM class of the scikit-learn library. The SVM kernel is set to rbf (radial basis function), with a penalty parameter C of 1.0 and Gamma set to ‘auto’. After training the SVM model, the cross_val_score function in the model_selection class of the scikit-learn library is used to perform 10-fold cross-validation to obtain performance evaluation metrics of the classifier, including accuracy, precision, recall, and F1-score. Throughout all the data processing procedures mentioned above, the StandardScaler function in the preprocessing class of the scikit-learn library is used to normalize the feature data.

## Results

This section primarily encompasses four experiments. The Evaluation of acoustic source biometric features aims to assess the extraction of optimal acoustic source features and evaluate the cough sound classification performance using single acoustic features by inputting them into an SVM classifier. The Evaluation of thermal source deep physiological features involves selecting the optimal feature extractor for extracting deep physiological features from thermal sources and evaluating the cough classification capability using single deep physiological features from infrared data by inputting them into an SVM classifier. The Evaluation of feature fusion combines the optimal features extracted from the previous two experiments using different fusion strategies. It evaluates the cough classification performance of the fused features by inputting them into an SVM classifier. Finally, the Evaluation of recognition speed and model size compares and evaluates the selected optimal methods’ recognition speed and model size from the previous three experiments.

### Evaluation of acoustic source biometric features

This experiment aims to evaluate the performance of the SVM-RFECV algorithm and select the optimal feature set as acoustic source biometric features from a pool of 29 cough sound features based on the experimental results. These selected features will then be inputted into an SVM classifier to assess the classification performance of single acoustic features for cough recognition. When comparing SVM-RFECV with other feature selection methods, we considered several factors that led to our choice of SVM-RFECV. SVM-RFECV offers an iterative process that eliminates irrelevant or redundant features, effectively reducing the impact of noise and enhancing the model’s robustness. Additionally, SVM-RFECV leverages the power of Support Vector Machines (SVMs), known for their ability to handle high-dimensional data and capture complex patterns. We achieve accurate feature selection by combining SVM with RFECV while maintaining computational efficiency. These advantages make SVM-RFECV a suitable choice for our specific application and contribute to its selection over alternative feature selection methods.

Regarding feature selection metrics, we employed the F1 score, which considers both precision and recall, providing a comprehensive assessment of the model’s performance. By evaluating the F1 score, we can effectively measure the trade-off between precision and recall, ensuring the selected features optimize the classification results.

**Fig 7:**
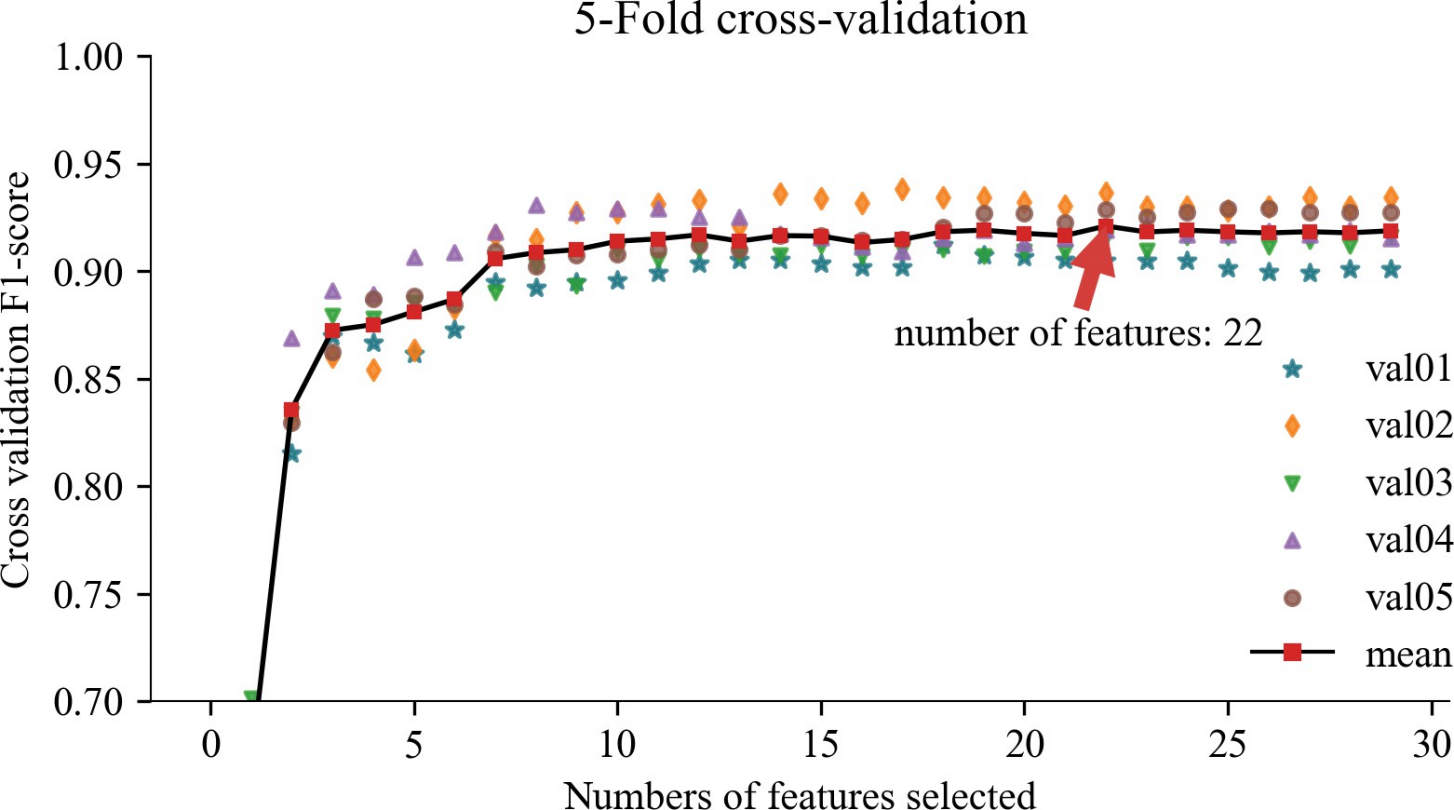
Evaluation results of acoustic source biometric features of 5-Fold.

**Fig 8:**
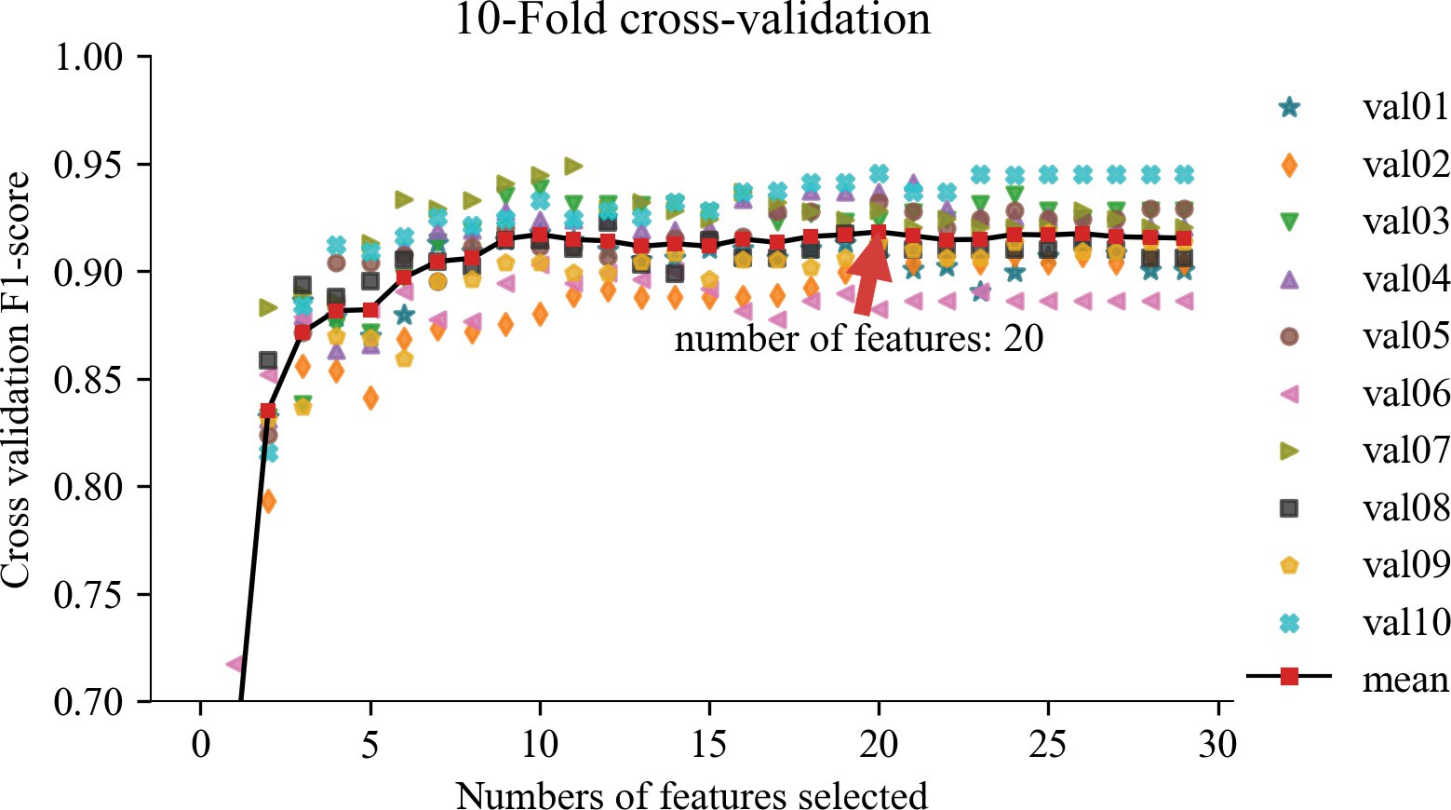
Evaluation results of acoustic source biometric features of 10-Fold.

**Fig 9:**
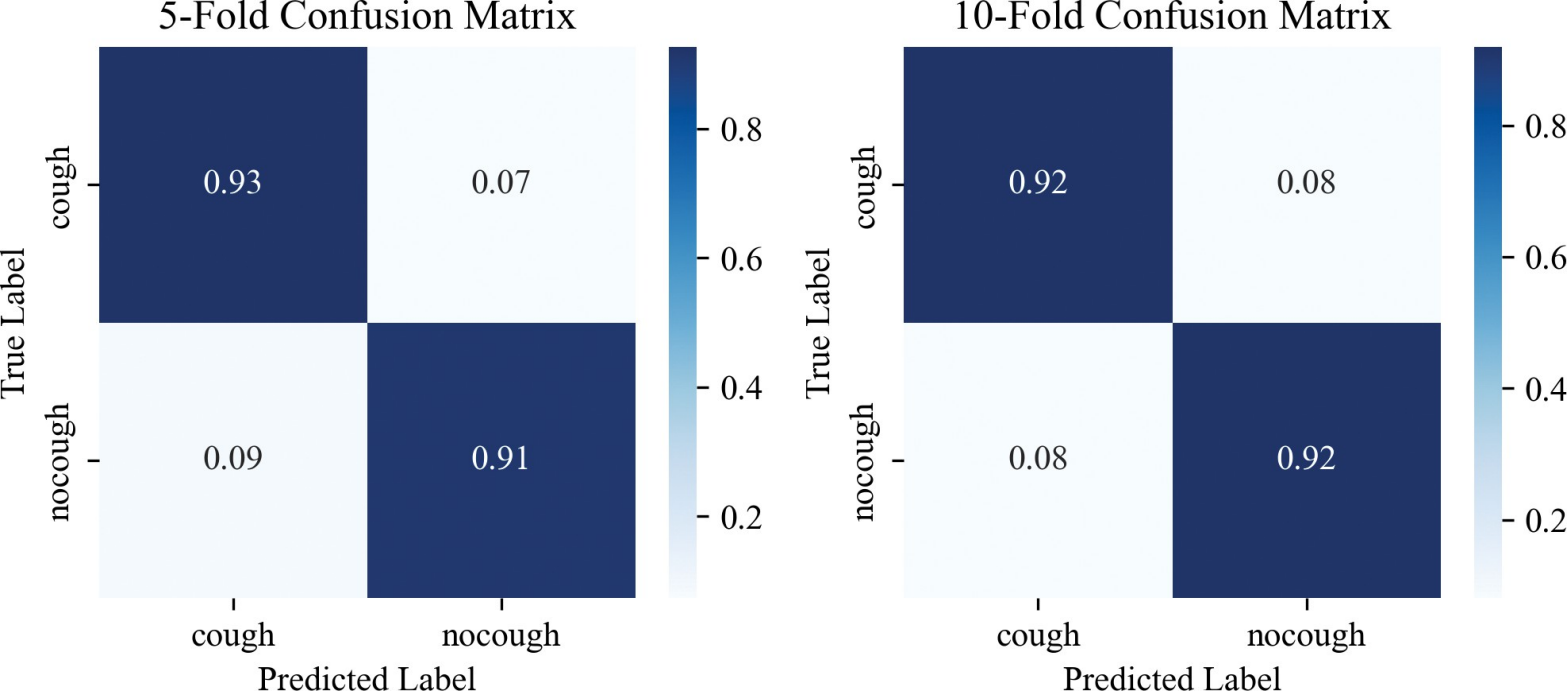
Evaluation results of acoustic source biometric features of confusion matrix results.

When employing SVM-RFECV for feature selection, we evaluated the number of features in the model and their respective performance using cross-validation test scores. Specifically, Figs 7 and 8 present the evaluation results using five-fold and ten-fold cross-validation, respectively. In Fig 7, we observe that an improved F1 score is achieved when utilizing eight features, and the F1 scores for both training and validation data tend to stabilize. Subsequently, the model’s accuracy gradually improves as the number of features increases. The optimal model evaluation metrics (Accuracy 92.13%, Precision 92.18%, Recall 92.05%, F1-score 92.10%) were reached when the number of features increased to 22. In Fig 8, the overall trend is consistent with Fig 7, and the optimal model evaluation metrics (Accuracy 91.84%, Precision 91.83%, Recall 91.89%, F1-score 91.82%) were reached when the number of features increased to 20. It can be seen that five-fold cross-validation is better than ten-fold cross-validation, which is consistent with the results of the analysis using the confusion matrix in Fig 9. This consistency across multiple evaluation facets solidifies the decision to adopt the 22 features corresponding to five-fold cross-validation as the optimal acoustic source biometric features for subsequent feature fusion. This judicious selection is underpinned by the superior performance and stability exhibited within the five-fold cross-validation framework.

### Evaluation of thermal source deep physiological features

**Fig 10:**
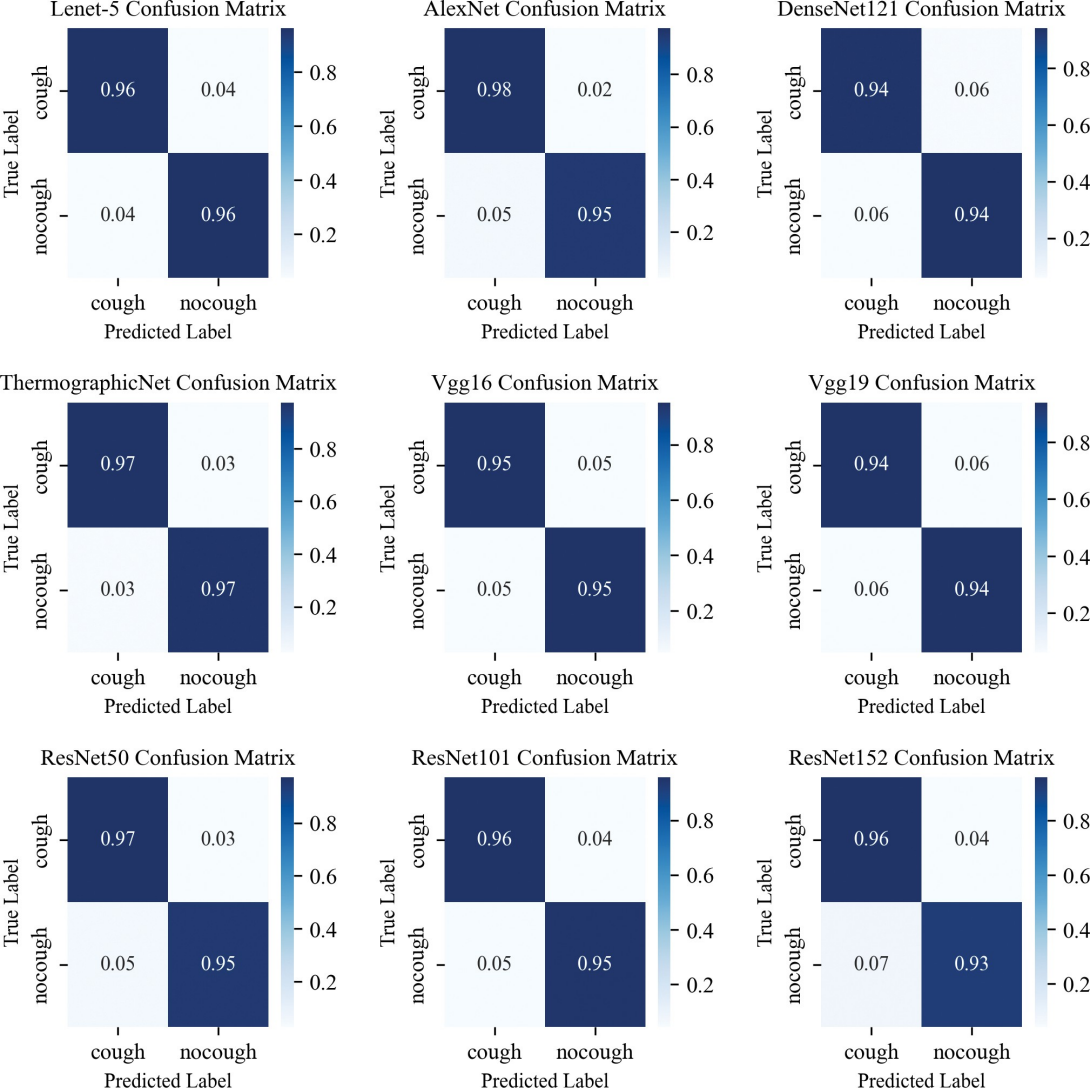
Confusion matrix results of thermal source deep physiological features.

This experiment aims to evaluate the capability of different CNN feature extractors in extracting deep physiological features from thermal sources in a given dataset. The optimal deep physiological features obtained from thermal sources will be inputted into an SVM classifier to assess the classification performance of single deep physiological features for cough recognition. The extraction ability of the fully connected layers of Lenet-5, AlexNet, DenseNet121, Vgg16, Vgg19, ResNet50, ResNet101, ResNet152, and ThermographicNet networks for deep physiological features was first evaluated, respectively. An infrared thermal image of the pig cough is taken as input, the most representative CNN network is selected as the feature extractor, and SVM is used as the classifier to accomplish the cough recognition task. Lenet-5 and AlexNet are prominent examples of shallow convolutional neural networks (CNNs), and ThermographicNet is a collection of the most representative CNN networks of Lenet-5 and AlexNet. VGG and ResNet are prominent examples of deep convolutional neural networks (CNNs). Shallow layers are characterized by their small size and portability, making them easy for application deployment. VGG is characterized by emphasizing deep layers with small filters, while ResNet introduces residual connectivity to address the challenge of vanishing gradients in deep networks. These models demonstrate the ability of deep CNNs to capture complex patterns and hierarchical representations. Then, using confusion matrix analysis, the feature vectors with the best performance are selected from the network’s fully connected layers with the most robust feature extraction capability as the thermal source deep physiological features. The performance evaluation metrics, including Accuracy, Precision, Recall, and F1-score, are used to evaluate the classification performance of SVMs using different CNN networks as feature extractors. The experimental results are shown in Table 2, and the corresponding confusion matrix results are shown in Fig 10.

**Table 2:** Comparative evaluation results of deep physiological features extraction.

CNN Feature Extractor		SVM Classifier Evaluation Metrics			
Name	FC Layer	Accuracy	Precision	Recall	F1-score
Lenet-5	FC1	95.41%	95.45%	95.41%	95.41%
	FC2	95.41%	95.45%	95.41%	95.41%
AlexNet	FC1	96.14%	96.22%	96.14%	96.13%
	FC2	95.82%	95.96%	95.82%	95.82%
DenseNet121	FC1	93.25%	93.33%	93.25%	93.25%
Vgg16	FC1	94.58%	94.63%	94.58%	94.58%
	FC2	94.58%	94.64%	94.58%	94.58%
Vgg19	FC1	93.16%	93.28%	93.16%	93.16%
	FC2	93.40%	93.50%	93.40%	93.39%
ResNet50	FC1	95.99%	96.06%	95.99%	95.99%
ResNet101	FC1	95.13%	95.19%	95.13%	95.12%
ResNet152	FC1	94.0%	94.15%	94.0%	94.0%
**ThermographicNet**	**FC1**	**96.80%**	**96.84%**	**96.80%**	**96.80%**
	FC2	96.68%	96.73%	96.68%	96.68%

The experimental results in Table 2 show notable differences between different feature extractors for cough classification. The average accuracy and F1-score of shallow CNN network feature extractors (LeNet-5, AlexNet, DenseNet121) are 95.21% and 95.20%, respectively. In contrast, the average accuracy and F1-score of deep CNN network feature extractors (Vgg16, Vgg19, ResNet50, ResNet101, ResNet152) are 94.41% and 94.40%, respectively. Our proposed ThermographicNet network feature extractor achieves an average accuracy and F1-score of 96.74%.

From the confusion matrix results in Fig 10, it is evident that, except for VGG19, the recognition performance of the fully connected layers in other network architectures is similar. FC1 consistently outperforms FC2 in classification performance. Furthermore, the ThermographicNet network proposed in this study demonstrates the best recognition performance across all four evaluation metrics. Based on the experimental results, we chose ThermographicNet network as the deep feature extractor and two fully connected layer features as the thermal source deep physiological features.

### Evaluation of feature fusion

**Fig 11:**
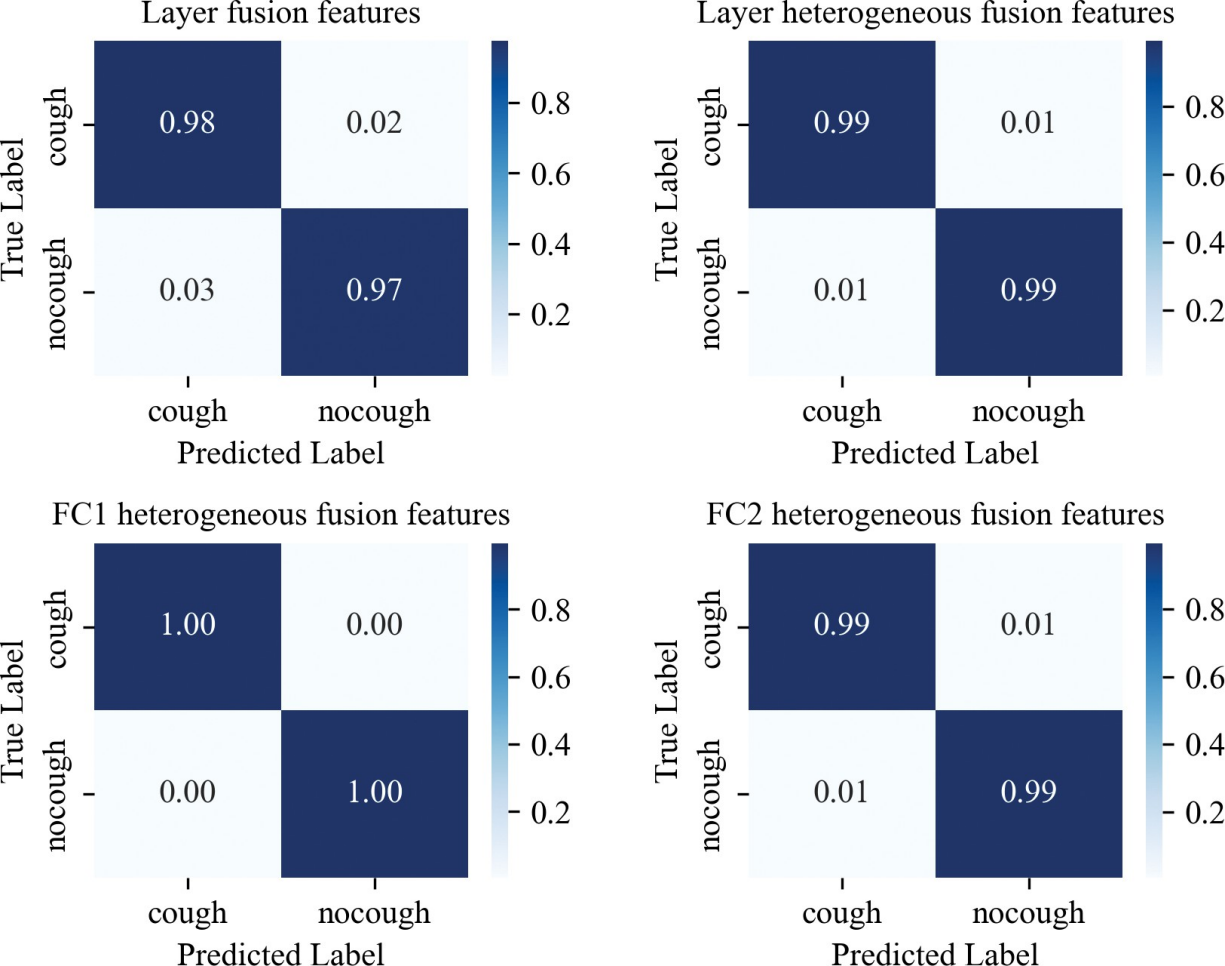
Confusion matrix results of fusion feature.

This experiment aimed to evaluate the ability of four different fusion strategies to classify pig coughing behavior to select the optimal fusion classification strategy. In Evaluation of acoustic source biometric features Section, the experiment selected the best acoustic set of 22 sound features as the acoustic source biometric features. In Evaluation of thermal source deep physiological features Section, the experiment was validated using our proposed ThermographicNet network as the feature extractor, obtaining the optimal classification performance based on the feature vectors from the FC1 layer’s output, which served as the thermal source deep physiological features. Following the fusion methods described in Feature fusion methods Section, the performance of four fusion methods, namely layer fusion, FC1 heterogeneous fusion, FC2 heterogeneous fusion, and layer heterogeneous fusion, were evaluated for cough classification, as presented in Table 3. The confusion matrix analysis results in Fig 11 demonstrate that different fusion strategies yield better results than a single feature. Three heterogeneous fusion methods outperform the homogeneous fusion method significantly. It is not surprising that combining biometric features with physiological features proves to be a practical approach, significantly improving cough recognition performance. The FC1 heterogeneous fusion method achieves an impressive classification accuracy and F1-score of 98.79%.

**Table 3:** Comparative analysis of pig cough recognition using different fusion strategies.

Features/Fusion features	SVM Classifier Evaluation Metrics			
	Accuracy	Precision	Recall	F1-score
acoustic source biometric features	92.13%	92.18%	92.05%	92.10%
FC1 thermal source deep physiological features	96.80%	96.84%	96.80%	96.80%
FC2 thermal source deep physiological features	96.68%	96.73%	96.68%	96.68%
layer fusion features	96.86%	96.89%	96.86%	96.86%
**FC1 heterogeneous fusion features**	**98.79%**	**98.80%**	**98.79%**	**98.79%**
FC2 heterogeneous fusion features	98.67%	98.68%	98.67%	98.67%
Layer heterogeneous fusion features	98.62%	98.62%	98.62%	98.62%

The three aforementioned experiments validate the proposed method as the optimal approach for cough recognition. Initially, the SVM-RFECV algorithm was employed to extract 22 features as acoustic source biometric features, which were then fed into an SVM classifier to evaluate the performance of using a single acoustic feature for cough recognition. Subsequently, the FC1 layer of the ThermographicNet network was utilized as a feature extractor to extract deep physiological features from thermal sources. These features were then evaluated using an SVM classifier to assess the performance of using thermal data alone for cough recognition. Finally, an early fusion technique combined the representative and significant features from both acoustic and thermal sources. The fused features were then inputted into an SVM classifier to evaluate the ability of heterogeneous fusion features in cough recognition. The experimental results demonstrate that the fused features outperform any single feature, thereby confirming the superiority and feasibility of the proposed method.

### Evaluation of recognition speed and model size

To further affirm the practical applicability of our proposed method in enhancing animal welfare, a comprehensive evaluation of the model’s recognition speed and size is imperative. An effective classification model should not only exhibit superior classification performance but also excel in recognition speed and model size, both of which are pivotal factors influencing the real-world deployment of the system. In field situations, computational efficiency and model size significantly impact deployment conditions, environmental settings, and overall costs. It is crucial to minimize computation time and achieve runtime acceleration for an efficient recognition process [48]. Therefore, we conducted a comparative assessment, evaluating ThermographicNet against other CNN feature extractors for deployment applications.

**Fig 12:**
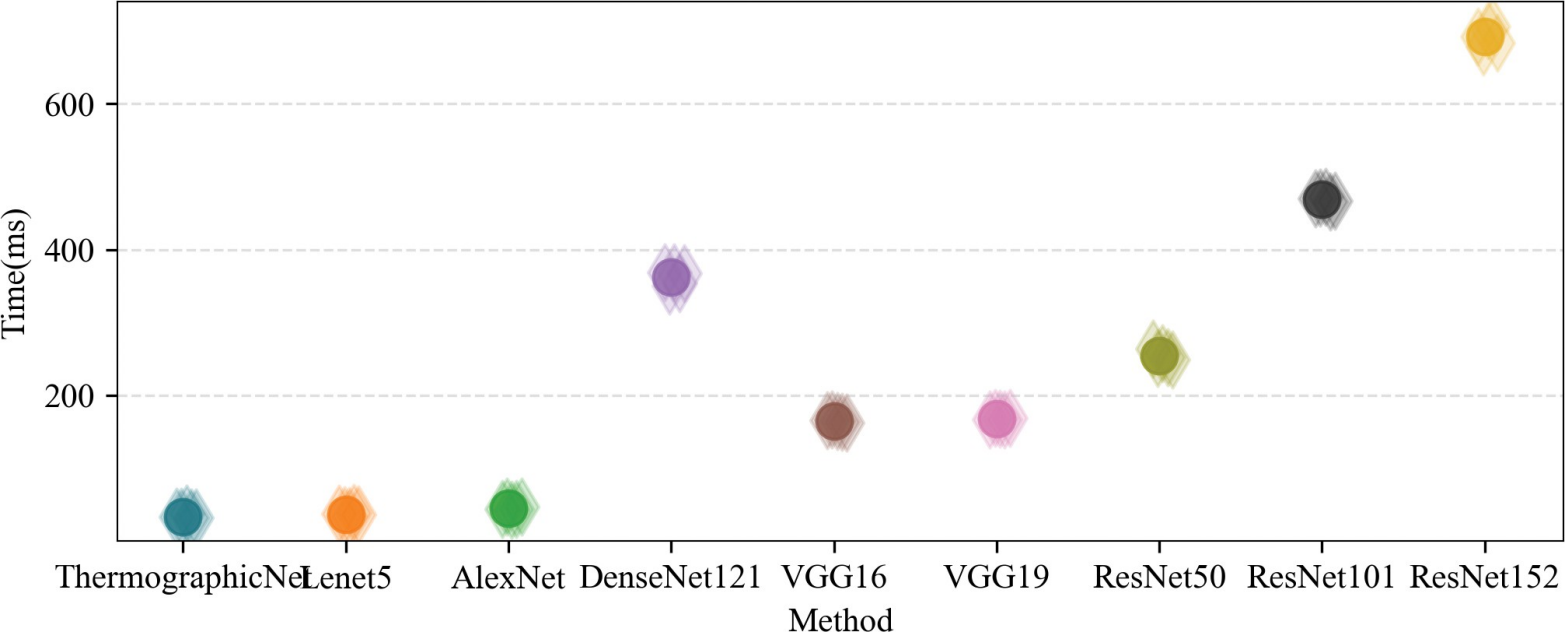
Comparison of computational time with different CNN networks using point plots.

A set of samples (5 samples) was randomly selected from our test set, each of which was computed five times on the computing platform without any additional load. The average execution time per sample and the average execution time per group were counted. The results are shown in Fig 12, a solid circle is the average time, and a hollow diamond is the individual time. From the experimental results. Our proposed Heterogeneous fusion method using ThermographicNet as a feature extractor has the fastest processing time, leading to a rapid increase in recognition speed. On average, the execution time of our proposed method is comparable to shallow CNNs, faster than all other deep CNNs, 4.7 times faster than the VGG family, and 13.9 times faster than the ResNet family.

**Fig 13:**
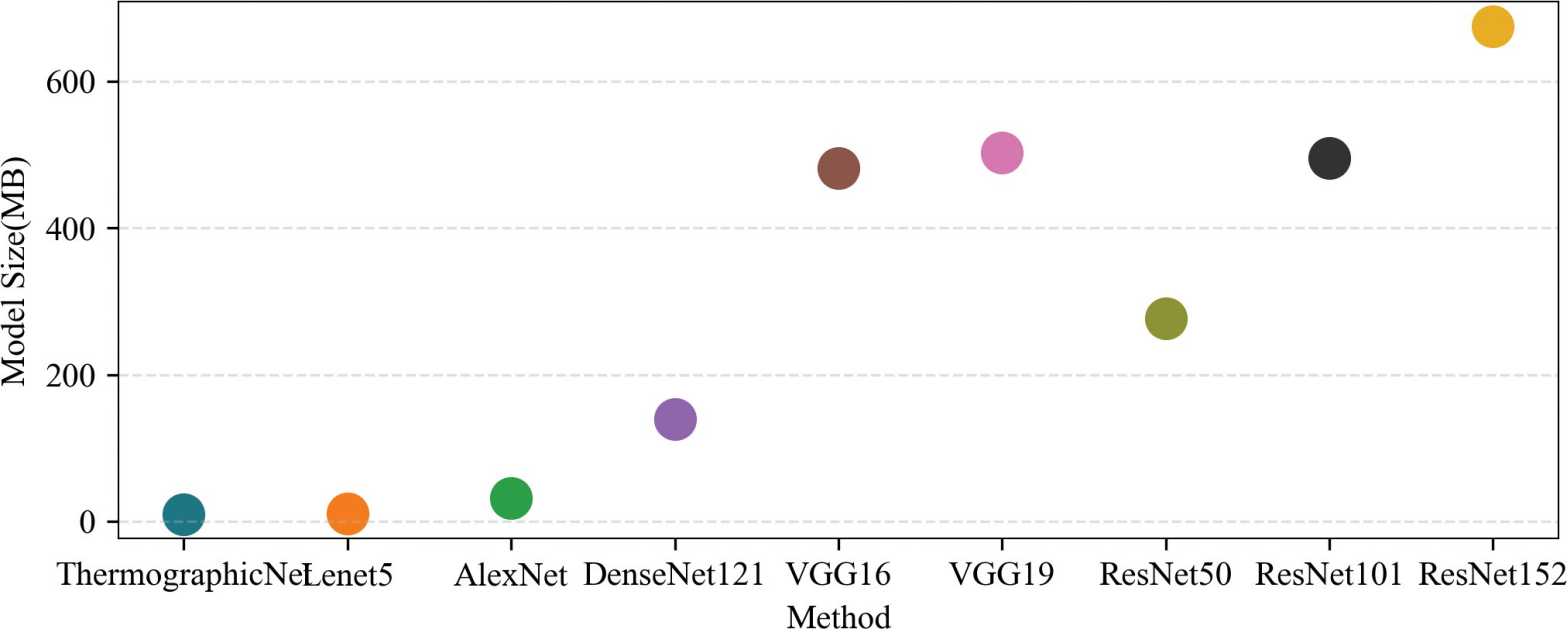
Comparison of model size with different CNN networks using point plots.

Finally, the model sizes are evaluated because they directly affect the deployment scenario [49], and the results are shown in Fig 13. Compared to the other eight models, the size of our model is comparable to the size of the shallow series model, our model size is 12.5% of the average size of the VGG family and less than 2.7% of the ResNet family. Therefore, in terms of model size cost, our model has a clear deployment advantage. It can be deployed and executed not only on central servers and edge servers but also on portable devices, enhancing its versatility and accessibility.

## Discussion

According to Figs 7 and 8, the acoustic source biometric feature set exhibits satisfactory results in pig cough recognition, but there still needs to be more potential for performance improvement. The curve indicates a trend of feature elimination, suggesting that incorporating additional features could enhance classification performance and expand the dimensionality of acoustic features [50,51]. However, the slope of the curve in Figs 7 and 8 implies that the performance gains from adding homogeneous acoustic features are increasingly challenging. To address this, we can enhance performance by exploring more diverse extended features and selecting a smaller, more refined feature set tailored specifically for pig cough recognition. Future research should explore additional features beyond the three domains of acoustics. Furthermore, extracting deep features from sound can enhance the recognition performance of individual audio samples [52], thereby determining the optimal set of acoustic features for pig cough sound recognition. Additionally, it is crucial to consider incorporating signal features beyond acoustics to overcome the performance bottleneck associated with single-feature analysis. Existing studies support these findings. For instance, Samson et al. [53] demonstrated that selecting representative features significantly improves classification accuracy in speech emotion recognition. Similarly, Saisanthiya et al.[54]and Raheel et al. [55] showcased the performance enhancement achieved by integrating heterogeneous features such as text or physiological signals in sound recognition tasks. Kakuba et al. [56] conducted an extensive exploration of modal and cross-modal modeling machine fusion methods, focusing on fusion studies involving sound and lexical semantics across various temporal, spatial, and semantic dimensions. Drawing inspiration from this work, future investigations into animal behavior could leverage a similar approach by integrating different modalities, including behavior, physiology, ecology, and genomics. This holistic perspective promises more practical and applied research outcomes in the realm of animal behavior studies. In summary, by leveraging a diverse range of extended features and carefully selecting a refined feature set, we can further enhance the performance of pig cough recognition. Future research should continue expanding acoustic features while considering incorporating additional signal features.

The experimental results presented in Table 2 clearly demonstrate the significant potential of thermal image depth features as a valuable tool for identifying pig coughs under field conditions. Infrared images have proven to be remarkably successful in recognizing cough patterns [57,58], with performance on par with current mainstream research focused on sound-based cough recognition. Notably, in the shallow CNN feature extractor, AlexNet (FC1) exhibited impressive performance, achieving an F1 score of 96.13%. Similarly, the deep CNN feature extractor, ResNet50 (FC1), demonstrated an outstanding performance with an impressive score of 95.99%. Evidently, shallow CNNs outperformed their deep counterparts in extracting essential features, while the inclusion of a fully connected layer had a limited impact on enhancing the classification performance. This outcome can be attributed to the characteristics of thermal image data, where shallow CNNs already capture a sufficient amount of information. This finding is in line with Ji et al. [16], which favours us to improve our model to obtain better future results. This indirectly validates the rationale behind leveraging the strengths of Lenet and AlexNet to design ThermographicNet, a deep physiological feature extractor tailored for thermal sources, as proposed in this paper. Additionally, Figs 12 and 13 further validate the superiority of our feature extractor in terms of its ability to extract features, processing speed, and model size.

The results presented in Table 3 demonstrate that the fusion of acoustic source biometric features and thermal source physiological deep features in a multi-source framework is an efficient and robust approach for pig cough recognition. However, when it comes to the homogeneous fusion of deep physiological features using the Layer fusion method, only a 96.86% F1 score was achieved. This is only slightly better than the 96.8% F1 score obtained with a singlethermal source physiological deep feature. Moreover, adding more homogeneous features and increasing computation and storage complexity did not significantly improve the classification performance. These findings align with previous research on homogeneous fusion in the field of speech [16,17], indicating that there is a performance bottleneck in homogeneous fusion.

On the other hand, three heterogeneous fusion methods, namely FC1 heterogeneous fusion, FC2 heterogeneous fusion, and layer heterogeneous fusion, demonstrated notable performance improvements and validated the complementary nature of heterogeneous features. Particularly, the FC1 heterogeneous fusion method achieved an astonishing 98.79% classification performance, which currently stands as the best result in cough recognition through feature fusion. This can be attributed to the fact that the shallow abstraction depth features derived from infrared data already effectively represent the physiological depth features of cough. Further, adding fully connected layers and fusing more homogeneous feature data contributed little to the classification. Conversely, fusing heterogeneous sound and infrared feature data, which represent biological and physiological signals, respectively, proved to be mutually beneficial and complementary. This insight suggests that future research on animal behavior recognition should consider a multimodal perception approach, incorporating tactile, auditory, and visual modalities.

In addition to achieving successful classification performance, the Heterogeneous fusion method proposed in this study demonstrated clear advantages in model size and recognition speed, as evidenced by the experimental results in Figs 12 and 13. This makes it suitable for deployment in real farm production environments, thereby improving animal welfare and enhancing early clinical diagnostic applications, especially for monitoring respiratory health in pigs.

Lastly, a recent analysis of the latest research on porcine cough recognition revealed that Yin et al. [14] achieved a high score of 99.2% by incorporating late fusion, specifically classifier fusion, in addition to feature fusion. This suggests that, in certain scenarios where time and space complexity requirements are not stringent, other fusion techniques beyond feature fusion can be considered to enhance cough recognition performance. It presents a potential research direction to explore.

**Fig 14:**
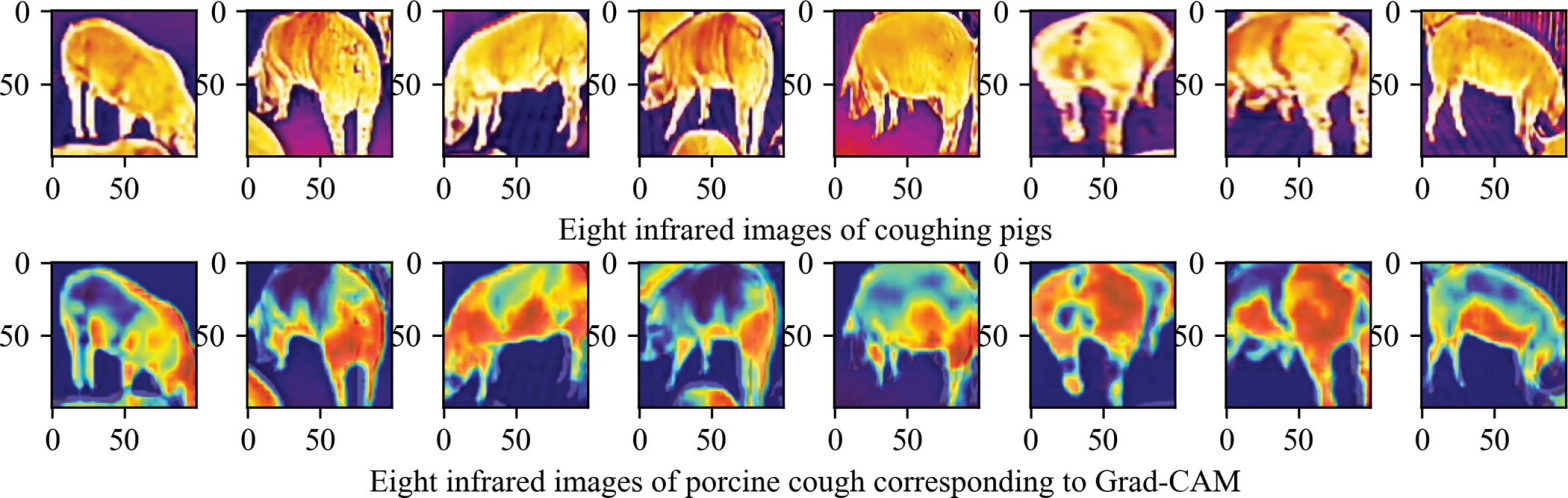
Gradient weighted class activation mapping of ThermographicNet.

One of the main challenges in training deep learning networks is the requirement for large amounts of labeled data, and a potential future solution could be adopting a weakly supervised learning paradigm [59]. Another challenge lies in the interpretability of the model, where the focus on the right features becomes more crucial than the overall accuracy. To delve into the interpretability of analyzing infrared physiological features, we employed the ThermographicNet model constructed in this study for an interpretability analysis using the Gradient Weighted Class Activation Mapping (Grad-CAM) technique. Eight infrared images of coughing pigs were randomly selected from the test set, and the resulting Grad-CAM maps are presented in Fig 14. As evident from Fig 14, the depth feature sites extracted by the ThermographicNet model predominantly include the mouth, nose, ear, and groin of the pig. These key sites are entirely interpretable from the standpoint of animal physiology and pathology. Coughing induced by upper respiratory infections is primarily characterized by elevated temperatures in the ear canal, mouth, nose, and groin. This is reflected in the infrared data, where the infrared features of these specific parts are more prominent, and our feature extractor adeptly captures these features. Therefore, not only is it feasible to conduct research on automatic respiratory health detection using depth features, but it also holds physiological interpretability for pigs. This stands as one of the principal contributions of this paper. Furthermore, extracting physiological deep features from thermal infrared images may necessitate additional equipment, technical support, and a more complex data acquisition environment. This could involve procuring thermal infrared imaging equipment and implementing intricate image processing and analysis techniques, thereby increasing the research difficulty and cost and limiting its applicability and generalizability. Therefore, in the actual deployment, full consideration should be given to the integrated application of the existing monitoring and other systems in the farm.

The merits of our methods are primarily geared towards intensive farms, aiming for non-contact and stress-free automatic detection of the respiratory health status of pigs. The objective is to achieve early detection and treatment, significantly reducing the workload on farm staff, improving work efficiency, cutting farming costs, boosting profitability, and ultimately enhancing animal welfare. The experimental results substantiate the advantages and viability of our approach. However, our method is susceptible to interference from anomalies such as persistent high-frequency noise in the environment, unexpected intrusion of heat generators, and equipment shielding, among others. Consequently, we must employ additional measures and techniques in data preprocessing and deployment implementation, leading to a certain increase in costs and performance fluctuations. To address the impact of spatial distribution on data quality [60], we have employed a data acquisition system with a cloud-side-end architecture for data acquisition. Relevant equipment is strategically deployed in designated locations, following a standardized production deployment approach. This ensures continuous 24-hour data collection, covering a spectrum of acoustic variations throughout the entire production process. Moreover, it helps mitigate the impact of thermal attenuation resulting from distance and ambient space, effectively dealing with the challenges posed by spatial differences. While various factors may influence method performance, the disparity between experimental results and actual deployment applications is negligible. Recent advancements in biological and physiological sensing technologies have proven beneficial for improving livestock health. In the future, we intend to enhance swine respiratory health monitoring conditions by developing an all-in-one machine that can enhance the quality of audio and thermal images collected within the barn.

## Conclusions

In this study, we propose a multimodal feature fusion approach that fuses acoustic source biometric features and thermal source physiological deep features in a multi-source classification framework to improve the recognition performance of cough in pigs. We enriched acoustic source biometric features by extracting thermal source deep physiological features from a shallow convolutional neural network. We utilized the complementary nature of different biological and physiological features to enhance cough recognition. Our study concludes that cough recognition using infrared images is effective in a swine barn environment, and the heterogeneous fusion method is more suitable for recognizing coughing behavior than the traditional acoustic homogeneous fusion. In the future, we can extend this study by applying other bioacoustic and physiological infrared samples for cough classification in field situations, which is essential for improving animal welfare and realizing smart animal husbandry.
